# Isolated cortical blindness without simultaneous neurological involvement in progressive multifocal leukoencephalopathy in a patient with human immune deficiency virus infection

**DOI:** 10.1186/1869-5760-3-3

**Published:** 2013-01-03

**Authors:** Vijay Ananth Jeyaraman, Sridharan Sudharshan, Ambika Selvakumar, Shika Bassi, Olma Veena Noronha, Poongulali Selvamuthu, Nagalingeshwaran Kumarasamy, Jyotirmay Biswas

**Affiliations:** 1 Medical Research Foundation, Sankara Nethralaya, No. 18, College Road, Nungambakkam, Chennai, Tamil Nadu, 600 006, India

**Keywords:** Cortical blindness, Progressive multifocal leukoencephalopathy, HIV infection

## Abstract

**Background:**

This is a case report of cortical blindness in a HIV-positive patient with progressive multifocal leukoencephalopathy (PML) without any other associated neurological dysfunction.

**Findings:**

Young HIV-positive patient presented to us with sudden profound visual loss. On examination and further investigation, we have diagnosed cortical blindness without any other focal neurological deficit due to PML.

**Conclusion:**

Our case highlights the fact that PML needs to be suspected in patients with HIV, presenting with cortical blindness even without any other focal neurological defect.

## Findings

### Introduction

Progressive multifocal leukoencephalopathy (PML) is a demyelinating disorder of the central nervous system (CNS) found in immune-deficient patients. It is caused by John Cunningham virus, a human polyomavirus (DNA virus) belonging to Papovaviridae group [1]. PML was originally described in patients with chronic diseases associated with compromised immune response or with immunosuppressive drugs [2,3]. However, the disease has been most frequently documented in patients with acquired immunodeficiency syndrome (AIDS) [4,5]. Studies estimate that prior to effective antiretroviral therapy, as many as 5% of people with AIDS eventually developed PML [6]. Usually, the outcome of patients of PML is poor; the disease is most often rapidly fatal with an expected progression to death within 6 months of symptoms [6,7]. Demyelination of the CNS is a consequence of virus-induced killing of oligodendrocytes, although the exact mechanism of cell death is unknown.

Characteristic presentations of PML are focal neurological deficits, generally without fever or headache. The progression of deficits leads to life-threatening disability and death over weeks to months. Neuroimaging plays a crucial role in the early diagnosis and longitudinal monitoring of functional integrity of the nervous system.

Since the central visual pathways and brainstem may be affected, a variety of neuro-ophthalmic signs and symptoms may manifest like visual symptoms, nystagmus, and cranial nerve and supranuclear gaze palsies [8,9]. Visual symptoms alone are infrequent in PML. We report a patient who presented with cortical blindness and without any other associated neurological dysfunction. Cortical blindness refers to visual loss in the presence of normal fundus and normal pupillary light reactions. It implies bilateral disease affecting the optic radiation or occipital lobes, most commonly caused by bilateral occipital lobe ischemia or trauma.

### Case report

A 34-year-old, recently diagnosed HIV-positive male presented to us with complaints of sudden painless decrease in vision in both eyes, 1 week before. On ocular examination, patient denied perception of light with indirect ophthalmoscope in dark room. Pupils were equal in size, and direct and consensual reflexes were normal. Ocular movements were full. Anterior segment slit lamp biomicroscopy and fundus examination with indirect ophthalmoscope were clinically within the normal limits. Systemic evaluation revealed no definite focal neurological deficits.

**Figure 1:**
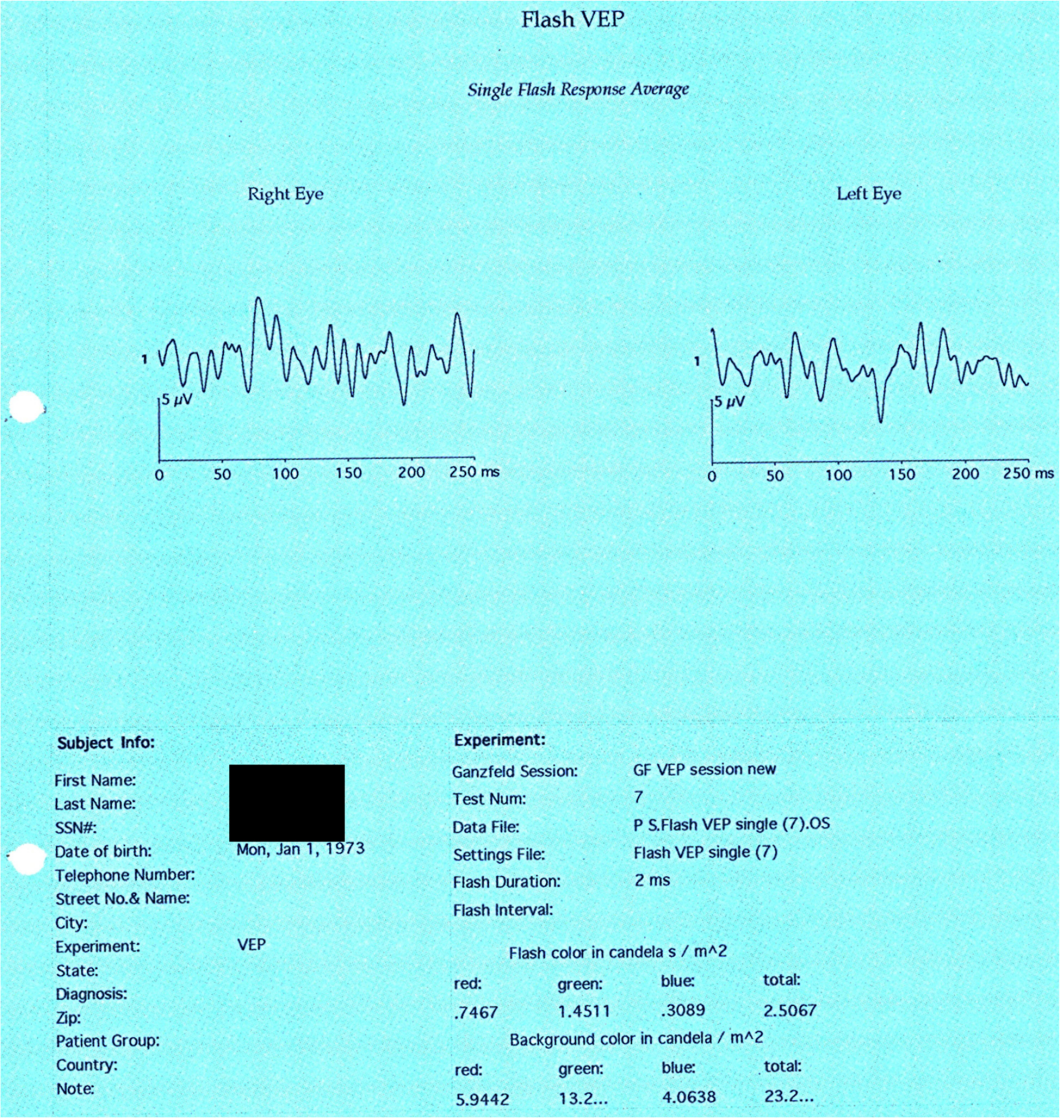
VEP showing a non-recordable response.

**Figure 2:**
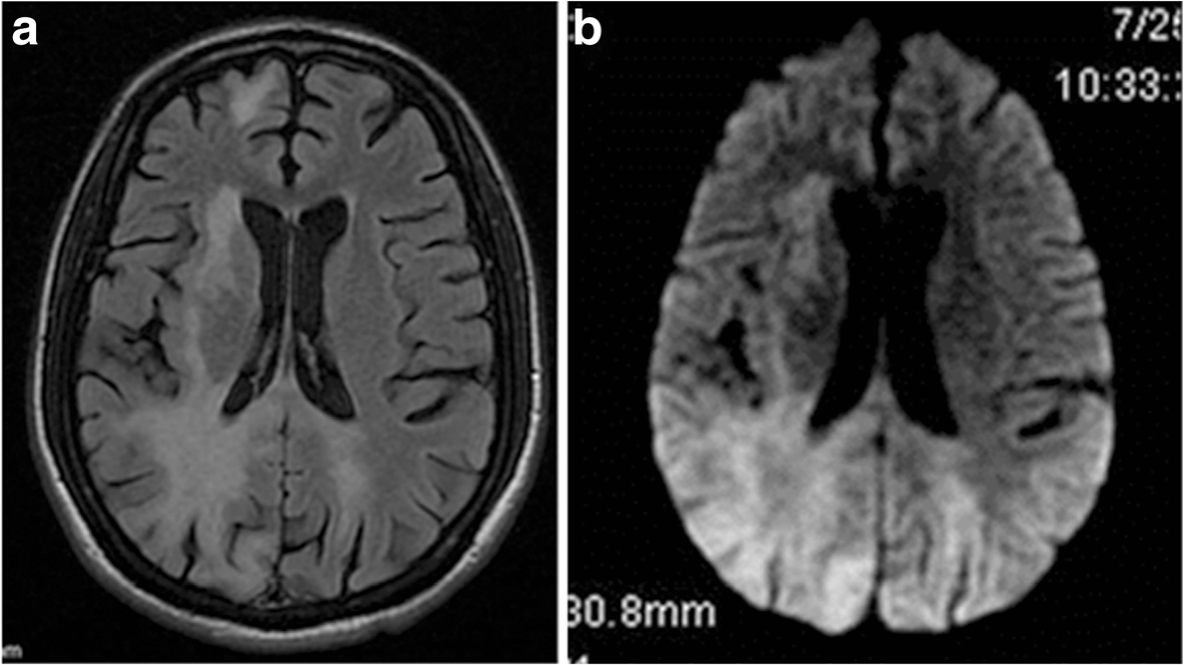
**Axial T2-Flair MRI and axial T2-Flair diffuse-weighted image. (a)** Axial T2-Flair MRI of the brain showing hyperintense signal intensity lesions bilaterally in the parieto-occipital white matter. **(b)** Axial T2-Flair diffuse-weighted image showing hyperintense lesions bilaterally in the parieto-occipital white matter.

A clinical diagnosis of cortical blindness was made. The patient underwent visual evoked potential (VEP) and magnetic resonance imaging (MRI). VEP showed non-recordable response (Figure 1). Axial T2-Flair MRI of the brain and orbit (Figure 2a) showed hyperintense signal intensity lesions bilaterally in the parieto-occipital white matter. Axial T2-Flair diffuse-weighted image (Figure 2b) showed hyperintense lesions bilaterally in the parieto-occipital white matter. MR spectroscopy showed elevated lactate and choline peak and reduction in the N-acetyl aspartate (NAA). These features were suggestive of demyelination. Optic nerve course, caliber, and signals were within the normal limits. Patient was evaluated systemically by the AIDS care physician and neurologist. Clinically, no cognitive or neurological deficits or other manifestations related to parieto-occipital and frontal disease were found. Patient CD4 count was 104 cells/mm^3^. Blood and CSF analyses to rule out other neurological illness commonly seen in patients with HIV such as cerebral tuberculosis, toxoplasmosis, *Cryptococcus* infection**,** or lymphoma were negative. The patient was initiated on highly active antiretroviral therapy (HAART) but refused inpatient treatment. Patient was brought to us few weeks later in an unresponsive state with terminal neurological illness and died within few hours.

### Discussion

PML has varied cerebral and brainstem manifestations. Most common initial manifestations are hemiparesis, mental deterioration, dysarthria, and dysphasia [2,5]. Although 20% to 30% of patients with HIV/AIDS can present with associated ocular manifestations at the time of diagnosis of PML [9], ocular involvement as the initial manifestation is rarely seen [3,10]. Patients presenting as hemianopic field defects and retrochiasmal field defects have been reported [5-9]. Most patients with PML have focal neurological deficits at the time of ocular manifestations.

Our patient presented with cortical blindness as a primary manifestation due to demyelination of bilateral occipital lobe involvement. There were no other systemic features attributable to PML. In spite of initiation on HAART, our patient died within few weeks of diagnosis.

HIV encephalopathy, cerebral tuberculosis, toxoplasmosis, lymphoma, *Cryptococcus* infection, and cerebral infarction need to be considered in the differential diagnosis in patients with HIV infection presenting with neurological illness [7]. Clinical features and neuroimaging were helpful in differentiating these conditions from PML as seen in our case.

MRI typically reveals multiple non-enhancing white matter lesions that may coalesce and have a predilection for the occipital and parietal lobes. The lesions show T2 hyper-intensity signals and diminished signal in T1-weighted images.

Recent improvements in newer imaging techniques like MR spectroscopy are replacing the older invasive methods for diagnosing PML. On MR spectroscopy, there is decreased NAA with significantly decreased NAA/creatinine ratio with increased choline and lactate peak [11] as noted in our patient. A diagnosis of PML in our case was made based on the presentation of a cortical blindness, rapidly progressive course of the disease, and MRI and MR spectroscopy scans consistent with PML.

Finsterer et al. [12] reported a patient presenting with cortical blindness along with bradydiadochokinesia and left ventricular hypertrabeculation/noncompaction. They also concluded that they may not have a causal relationship. Rickards and Shepherd also reported a patient with cortical blindness as the initial manifestation in PML [10]. Unlike our case, his patient had a progressive tunnel vision with associated neurological signs such as hemiparesis. Cortical blindness with PML has always been reported in the literature to be associated with other neurological deficits [9]. In our patient, cortical blindness was the only presenting feature and had no other associated neurological dysfunction.

### Conclusion

Our case highlights the fact that PML needs to be suspected in patients with HIV, presenting with cortical blindness even without any other focal neurological deficits. This case also illustrates the need of a prompt diagnosis in order to treat the patient as soon as possible considering the rapid evolution of the disease. We had not come across, after Medline search, any such report of a patient with HIV presenting with cortical blindness as the presenting feature of PML without other neurological deficits.

## Ethics

No ethical issues were involved in this case and also this case was presented, in our ethical committee.

## Competing interests

The authors declare that they have no competing interests.

## Authors’ contributions

VA /SS /OVN/PS- were directly involved in the patient management. VA/SS- Collection material for the case report, Documentation. VA/SS-written the Case report SA/KN/JB-Overview of the case report All authors read and approved the final manuscript.

## References

[B1] Manji H Miller RF Progressive multifocal leucoencephalopathy: progress in AIDS era J Neurol Neurosurg Psychiatry 2000 69 569 571 10.1136/jnnp.69.5.569 11032602 PMC1763420

[B2] Astrom KE Mancall EL Richardson EP Jr Progressive multifocal encephalopathy: a hitherto unrecognized complication of chronic lymphocytic leukemia and Hodgkin's disease Brain 1958 81 93 111 10.1093/brain/81.1.93 13523006

[B3] Appen RE Roth H Progressive multifocal leukoencephalopathy. A cause if visual loss Arch Ophthalmol 1977 95 656 659 10.1001/archopht.1977.04450040122019 849189

[B4] Stoner GL Ryschkewitsch CF JC Paovirus larger tumour (T)-antigen expression in brain tissue of acquired immune deficiency syndrome (AIDS) and non-AIDS patients with progressive multifocal leukoencephalopathy Proc Natl Acad Sci USA 1986 83 2271 2275 10.1073/pnas.83.7.2271 3008157 PMC323274

[B5] Berger JR Kaszovitz B Progressive multifocal leukoencephalopathy associated with human immunedeficiency virus infection. A review of the literature with a report of sixteen cases Ann Intern Med 1987 107 78 87 3296901 10.7326/0003-4819-107-1-78

[B6] Post MJD Yiannoutsos C David S Booss J Clifford DB Cohen B Progressive multifocal leukoencephalopathy in AIDS: are there any MR findings useful to patient management and predictive of patient survival AJNR Am J Neuroradiol 1999 20 1896 1906 10588116 PMC7657792

[B7] Hurley RA Ernest T Khalili K Valle LD Simone IL Taber KH Identification of HIV associated progressive multifocal leucoencephalopathy J Neuropsychiatry Clin Neurosci 2003 15 1 6 10.1176/appi.neuropsych.15.1.1 12556565

[B8] Wein F Francis GS Gans MS Connolly WE Burnier MN Jr Neuro-ophthalmic findings in progressive multifocal leukoencephalopathy Can J Ophthalmol 1998 33 5 270 275 9740956

[B9] Ormerod LD Rhodes RH Ophthalmologic manifestations of acquired immune deficiency syndrome-associated progressive multifocal leukoencephalopathy Ophthalmology 1996 103 899 906 8643245 10.1016/s0161-6420(96)30589-7

[B10] Rickards C Shepherd DI Cortical blindness in a 35-year-old man Postgrad Med J 1996 72 846 249 251 10.1136/pgmj.72.846.249 8733542 PMC2398431

[B11] Iranzo A Moreno A Pujol J Fabregas JM Domingo P Molet J Ris J Cadafalch J Proton magnetic resonance spectroscopy pattern of progressive multifocal leukoencephalopathy in AIDS J Neurol Neurosurg Psychiatry 1999 66 520 523 10.1136/jnnp.66.4.520 10201428 PMC1736302

[B12] Finsterer J Stöllberger C Coulibaly-Wimmer M Noncompaction in a HIV-positive with cortical blindness as initial manifestation of progressive multifocal leucencephalopathy Int J Cardiol 2011 149 1 e4 e7 10.1016/j.ijcard.2009.03.046 19328566

